# Spatial distribution and clusters of pancreatic cancer mortality in Shandong Province, China

**DOI:** 10.1038/s41598-019-49357-w

**Published:** 2019-09-09

**Authors:** Fan Jiang, Jie Chu, Xianxian Chen, Jiyu Zhang, Zhentao Fu, Jiandong Sun, Zilong Lu, Xiaolei Guo, Aiqiang Xu

**Affiliations:** 1 The Department for Chronic and Non-communicable Disease Control and Prevention, Shandong Center for Disease Control and Prevention, Jinan, China; 2 School of Public Health, Queensland University of Technology, Queensland, Australia

**Keywords:** Cancer epidemiology, Cancer epidemiology

## Abstract

This study aimed to explore the geographic distribution and risk clusters of pancreatic cancer mortality from 2011 to 2013 in Shandong, China, and to detect the differences between urban and rural areas. Our data were obtained from the Shandong Death Registration System (SDRS) and were adjusted according to the underreporting level. The distribution of mortality was displayed with GIS-based maps at the county level. The results showed an increasing trend in pancreatic cancer mortality from the western region to the eastern region of Shandong. Additionally, four significant risk clusters were detected, and the most likely cluster was focused in the northeastern and northern regions. Urban-rural differences in the mortality distribution and risk clusters were also detected. In conclusion, our study identified pancreatic cancer mortality clusters in Shandong in urban and rural areas; these results can contribute to the development of effective and targeted strategies to control pancreatic cancer in different areas.

## Introduction

Pancreatic cancer is one of the most fatal malignancies with approximately 338,000 new cases and 330,000 deaths per year. It is the 7^th^ leading cause of death from cancer worldwide^1,2^. In China, according to the National Central for Cancer Registry (NCCR), the mortality rate of pancreatic cancer in 2011 was 5.40 per 100,000, ranking 6^th^ among all cancer deaths^3^.

Pancreatic cancer mortality has not decreased over the past few decades like most other common cancers have^4,5^. In addition, it is expected that death due to pancreatic cancer will become the second leading cause of cancer death by 2030^6^. A Chinese study found that pancreatic cancer mortality increased from 1991 to 2000 and may reach its highest peak in the future^7^. Shandong Province is one of the fastest growing provinces for pancreatic cancer morbidity and mortality in China. Pancreatic cancer ranked 7^th^ in deaths from malignant tumors in Shandong from 2011 to 2013^8,9^.

Some studies have shown that pancreatic cancer mortality in urban areas of China is higher than that in rural areas^3,10,11^. One survey in 2010 also reported a higher mortality rate in the eastern region than in the central (1.3 times higher) and western region (1.5 times higher)^12^. Therefore, it is well evidenced that there are distinct differences in the geographic distribution of pancreatic cancer, and the mortality rates in urban areas and in the eastern region may be higher than those in rural areas and central and in the western region^10–12^.

Geographical differences in pancreatic cancer rates may exist among different administrative units in urban or rural areas. Monitoring cancer-related data in smaller units may be an effective means of identifying clear regional disparities^13^. Spatial statistical analysis is a demonstrated method for exploring spatial disease clusters of any size, including those in some smaller units^14,15^.

One previously published article explored the geographic distribution and clusters of breast cancer mortality based on an entire population at the county level in Shandong, China^16^. Using the same research method and statistical analysis, our study was conducted to examine the geographic distribution and risk clusters of pancreatic cancer mortality in Shandong, and to detect the differences between urban and rural areas.

## Materials and Methods

### Data collection

Since 2010, Shandong Province has maintained a comprehensive death registration report, which monitors and collects population data from142 counties (districts). We extracted data on pancreatic cancer death, which was coded as C25 according to the 10^th^ Reversion of International Classification of Diseases (ICD-10), in the Shandong Death Registration System (SDRS)^17^. A study conducted in Shandong estimated an overall underreporting of 21.5% in the SDRS from 2011 to 2013^18^. The level of death underreporting was likely to have large inter-regional differences, thus reducing the comparability of mortality between regions. For meaningful comparisons between areas and populations, we adjusted the mortality rates based on the underreporting levels. This study used the capture-mark-recapture (CMR) method to adjust the data from a population-based underreporting survey from 2011 to 2013 in Shandong Province. A study titled “Assessment of the underreporting rate of cause of death data in Shandong Province using the capture-mark-recapture method” introduced this method in detail; it stated “ It is hypothesized that M independent individuals in a randomly acquired sample from the targeted population with N individuals are marked and released to the original population. Then, another random sample is acquired with n independent individuals from the same targeted population to identify the number of marked individuals (m). An unbiased formula was used to estimate the size of the targeted population according to the two independent samplings”^19^. According to the principle of this method, this study assumed that the total number of pancreatic cancer deaths (targeted population) was N, M and n represented the number of deaths in two independent death events in the registration system and the underreporting survey, respectively, and m represented the number of repeated deaths among the two sources. The total number of deaths was estimated based on the unbiased formula. The population data were extracted from the Shandong statistical yearbook. In this study, the number of pancreatic cancer deaths was 8371 before the underreporting adjustment, and the mortality rate was 2.9 per 100,000 people; however, after the underreporting adjustment, the number of deaths was 11468, and the mortality rate was 4 per 100,000 people.

We used the same urban-rural classification method as reported in the Methods section of the article titled “Female Breast Cancer Mortality Clusters in Shandong Province, China: A Spatial Analysis”, which stated “There are six levels in the Chinese administrative system: the national, provincial, prefectural, county, town, and village level. In Shandong Province, there are 17 prefectures comprising 142 county-level units (counties or districts). Each county-level unit usually includes two types of town-level units: a sub-district and township. We defined the urban population as people living in sub-districts, and the rural population as people living in townships. There are no rural townships in 17 urban districts, and thus, only the urban population was defined in such districts. For 5 counties with predominantly rural populations, the data of the sub-district populations were unavailable, and we treated the population of the entire counties as rural populations. In total, we defined 262 sub-county level units for assessment. Among them, 137 were urban units and 125 were rural units. Of these, 4 units, including 3 urban units and 1 rural unit, had small populations and had no pancreatic cancer deaths during the study period; thus, we classified their mortality as zero”^16^.

### Statistical analysis

The average reported mortality rate (ARMR) of pancreatic cancer was obtained by calculating the ratio of the total number of deaths in each county (district) to the corresponding total population from 2011 to 2013. It is the temporal aggregation of deaths over a 3 year period that is expected to reduce mortality variations in small areas and populations. Standardization was necessary when comparing several populations with different age structures because age had such a powerful influence on cancer incidence and mortality. The age-standardized rate (age-adjusted rate) was a summary measure of a rate that a population would have if it had a standard age structure. The age structure was divided into 19 age groups, including 0~, 1~, 5~, 10~, …, 85~. This study used the population composition of 1964 Chinese National Census as the standard population to calculate the age-adjusted and crude mortality.

$${\rm{Age}}-{\rm{adjusted}}\,{\rm{rate}}\,\frac{\sum (Standard\,population\,in\,corresponding\,age\,group\ast age-specific\,rate)}{\sum Standard\,population}$$

Age was adjusted in the total population and in the urban/rural populations to explore its effects on pancreatic cancer clusters. The statistical significance of clustering was based on the Monte Carlo hypothesis test, which compared the likelihood ratio test statistic of the observation data set with the test statistic of 999 random data sets generated under the non-cluster null hypothesis^20^. The basic idea of spatial scan statistics was to perform traversal scans in time, space or space-time by setting the scan window to find statistically significant high-risk areas. With the moving window method, a dynamic circular window was established in the study area to scan for and diagnose pancreatic cancer deaths. For each change, the difference in pancreatic cancer mortality between the inside and outside of the scan window was calculated and tested with the log likelihood ratio (LLR). We set a 50% risk population as the size of the maximum spatial scan window. The most likely cluster was defined as the set of connected regions that attained the maximum LLR. Secondary clusters that rejected the null hypothesis but did not overlap with the most likely cluster were also reported.

The mortality rate and age-adjusted rate were calculated with Stata13.1 (Stata Corporation, College Station, TX, USA). The county-level mortality distribution maps and risk clusters were drawn using ArcGIS 10.2 (ESRI Inc., Redlands, CA, USA)^21^. Spatial clustering analysis was performed to identify the high-risk areas (clusters) by SaTScan 9.5 (NCI, Boston, MA, USA)^22^. All reported probabilities (*p* values) were two-sided, and values less than 0.05 were considered statistically significant.

### Study approval

In this study, data we used was the monitoring data reported to China every year, which was approved by the health administrative department. We acquired data through official surveillance of the Shandong Death Registration System. So we think no informed consent statement is required. The entire research protocol was approved by the Ethics Committee of Preventive Medicine of the Shandong Center for Disease Control and Prevention in 2013 (approval number 2013020). Data collection and analysis methods were carried out in accordance with relevant guidelines and regulations.

## Results

### Descriptive analysis

From 2011 to 2013, a total of 11468 pancreatic cancer deaths occurred in Shandong, with an ARMR of 4.00 per 100,000 people (crude rate). According to the composition of 1964 standard population, the age-adjusted rate was 1.53 per 100,000 people. Pancreatic cancer accounted for 2.28% of all malignant tumors and 0.52% of all deaths. The crude mortality rate at the county level ranged from 0.22 to 15.39 per 100,000 people (the age-adjusted rate ranged from 0.09 to 4.44 per 100,000 people).

**Figure 1:**
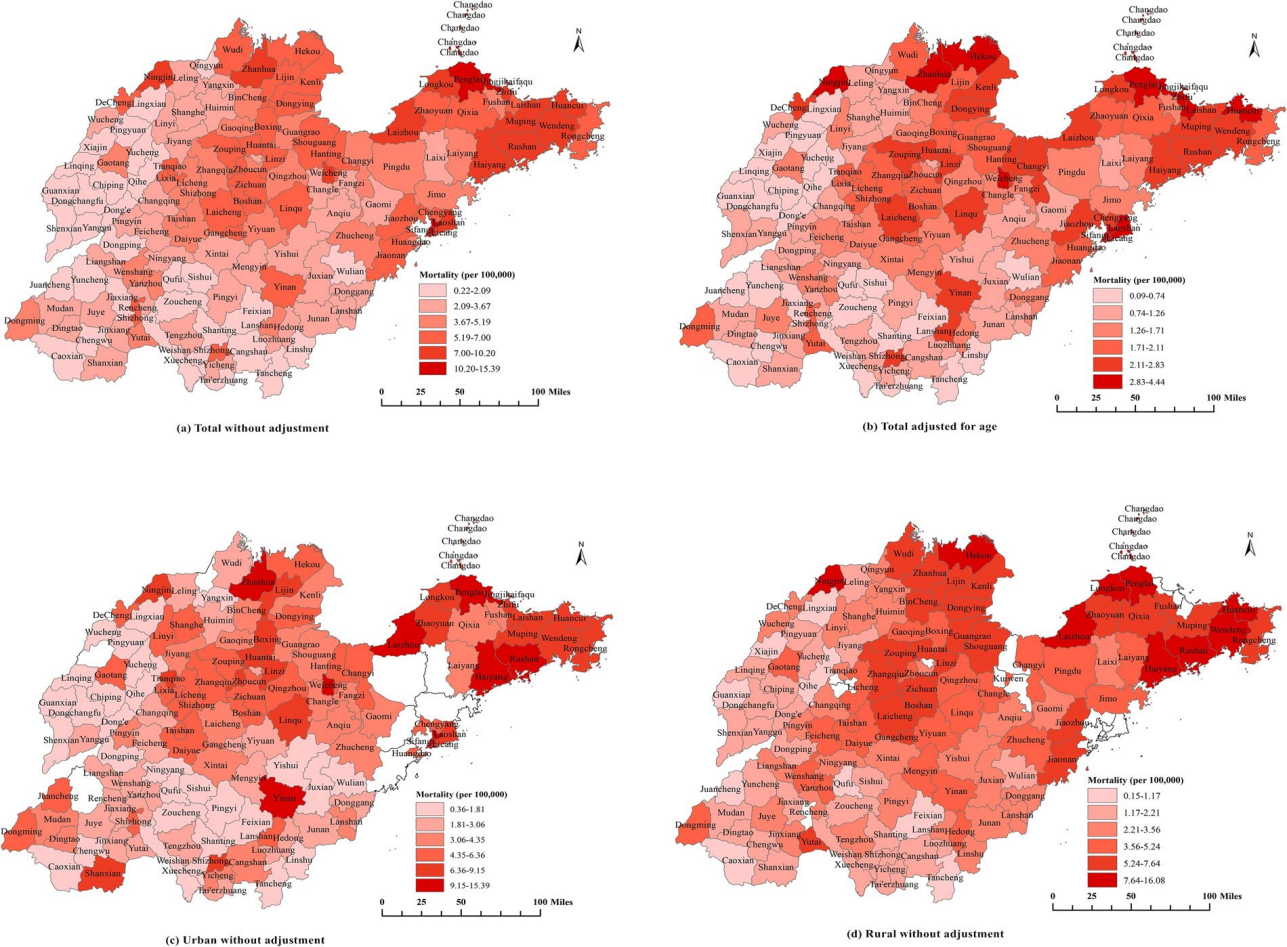
Geographic distribution of pancreatic cancer mortality at the county level in Shandong on Urban/Rural Status for the years 2011 to 2013. (**a**) Total mortality without adjustment; (**b**) Total mortality adjusted for age; (**c**) Urban mortality without adjustment; (**d**) Rural mortality without adjustment. Note: the red scale represents the level of mortality rate and the redder the color, the higher the mortality rate.

Figure 1(a,b) showed the geographic distribution of the crude mortality and age-adjusted mortality from pancreatic cancer, respectively; the red scale represents the level of mortality rate and the redder the color, the higher the mortality rate. Overall, both the crude mortality and the age-adjusted mortality were higher in the eastern region than in the western region. Figure 1(a) showed that areas with high mortality rates were mainly concentrated in some counties in the eastern region, including most of the counties in Yantai, Weihai and Qingdao cities; some counties of the northern region, including most of the counties in Dezhou and Binzhou cities; northwest of Jinan city; some counties in Zibo city; and Weicheng district in Weifang city. However, mortality rates in the central and western regions were relatively low. Figure 1(b) showed that areas with relatively high age-adjusted mortality rates included Weihai, Yantai, Qingdao, Dongying, Zibo, Weifang and Dezhou cities. According to the above, after adjusting for age, some new high-mortality areas were identified, such as Dongying city.

Figure 1(c,d) showed the distribution of pancreatic cancer mortality in urban and rural areas, respectively. The mortality rates in urban areas were slightly higher than those in rural areas. In urban, areas with high mortality rates were mainly located in the eastern, northern and central regions; the highest mortality rate was in Sifang district in Qingdao city, and the lowest mortality rate was in Ling County in Dezhou city (Fig. 1c). In rural, areas with high mortality rates were focused in the eastern, northern and central regions; the highest mortality rate was in Changdao county of Yantai city, and the lowest mortality rate was in Ling county of Dezhou city (Fig. 1d).

### Spatial scan statistics analysis

**Figure 2:**
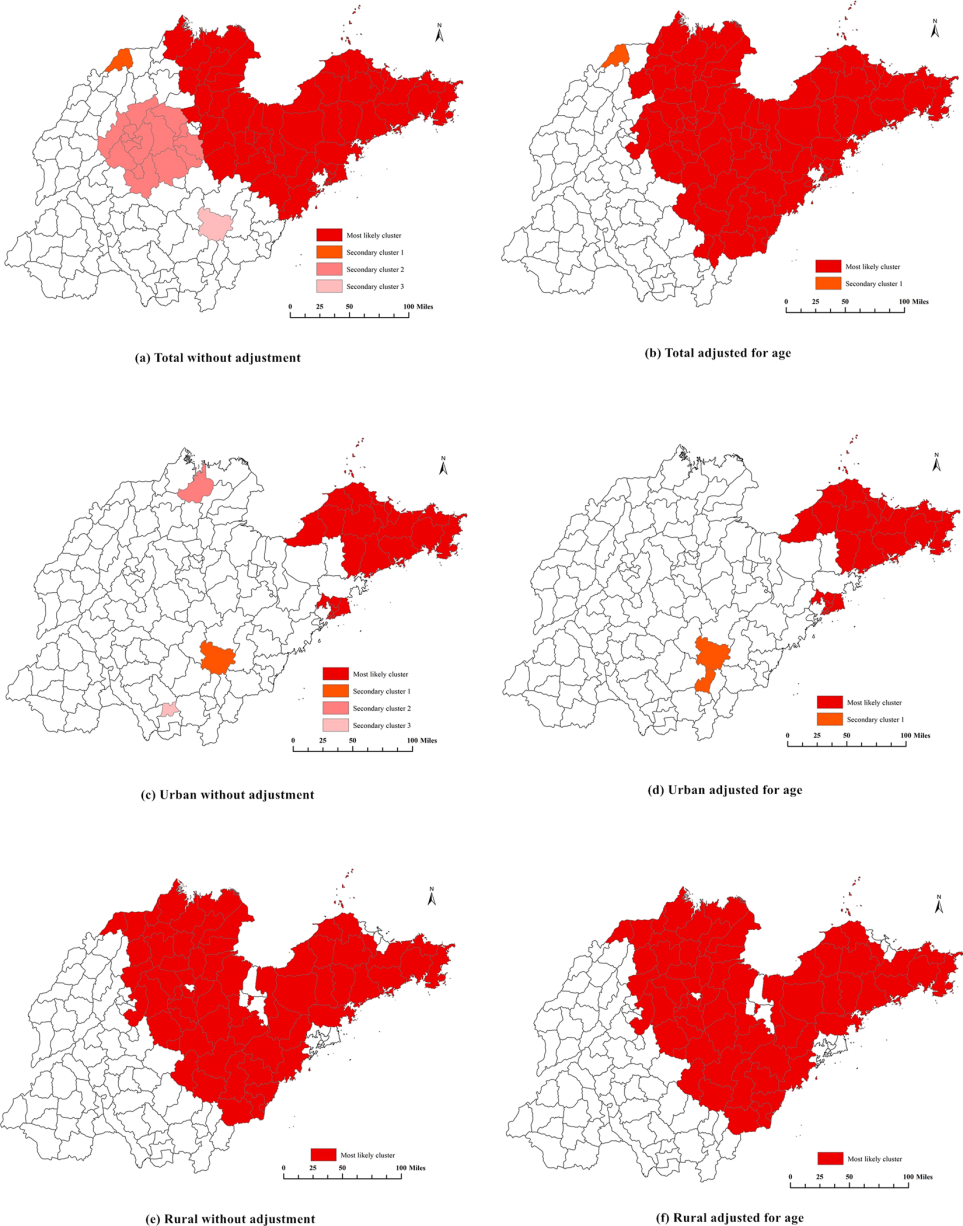
Pancreatic cancer mortality clusters at the county level in Shandong for the years 2011 to 2013 using the spatial scan statistical analysis. (**a**) Total without adjustment (most likely cluster, secondary cluster 1, 2 and 3); (**b**) Total adjusted for age (most likely cluster and secondary cluster 1); (**c**) Urban without adjustment (most likely cluster, secondary cluster 1, 2 and 3); (**d**) Urban adjusted for age (most likely cluster and secondary cluster 1); (**e**) Rural without adjustment (only one most likely cluster); (**f**) Rural adjusted for age (only one most likely cluster).

The spatial scan statistics analysis revealed four significant clusters of pancreatic cancer mortality in Shandong (Table 1 and Fig. 2a). The most likely cluster covered all northeastern regions and some counties in the northern region, for a total of 52 counties (districts), and the relative risk (RR) of pancreatic cancer death in this area was 2.18 times higher than that of the other areas. The first secondary cluster only covered Ningjin county in Dezhou city, where the RR was 2.28 times higher than that of the other areas. The second secondary cluster covered 16 counties (districts) including Jinan city, Tai’an city, and Zibo city, and the RR was 1.20 higher than that of the other areas.

**Table 1:** Results of the spatial scan analysis of pancreatic cancer mortality at the county level in Shandong for the years 2011 to 2013.

Cluster	County amount	Cases	Expected	Annual case/100000	RR	LLR	P value
***Total without adjusted for age***							
Most likely cluster*	52	5769	3637	6.3	2.18	850.27	<0.01
Secondary cluster 1	1	128	56	9.1	2.28	33.47	<0.01
Secondary cluster 2	16	1480	1259	4.7	1.20	20.74	<0.01
Secondary cluster 3	1	161	111	5.8	1.46	10.08	<0.01
***Total adjusted for age***							
Most likely cluster	78	2815	2146	2.0	1.90	211.69	<0.01
Secondary cluster 1	1	54	21	3.8	2.57	17.89	<0.01
***Urban Without adjusted for age***							
Most likely cluster	23	1791	982	9.1	2.31	358.99	<0.01
Secondary cluster 1	1	61	25	12.3	2.49	18.97	<0.01
Secondary cluster 2	1	38	13	14.2	2.86	15.11	<0.01
Secondary cluster 3	1	80	44	9.1	1.84	12.10	<0.01
***Urban adjusted for age***							
Most likely cluster	23	624	383	3.2	1.94	83.92	<0.01
Secondary cluster 1	2	79	42	3.6	1.91	13.10	<0.01
***Rural without adjusted for age***							
Most likely cluster	65	4488	3150	5.0	2.32	549.81	<0.01
***Rural adjusted for age***							
Most likely cluster	65	1547	1151	1.7	1.95	131.13	<0.01

Note *: “Most likely cluster” was defined when the maximum log likelihood ratio (LLR) with statistical significance was detected by Monte Carlo simulation in spatial analysis; the other LLR values with statistical significance were identified as “Secondary cluster”. The relative risk (RR) was the ratio of the mortality inside a cluster area to the mortality outside a cluster area.

The third secondary cluster included only one county (Yinan county in Linyi city) with a RR of 1.46. After adjusting for age, there were two significant clusters detected (Table 1 and Fig. 2b). The most likely cluster covered all the counties in the northeastern region and most of the counties in the northern, central and southeastern regions, for a total of 78 counties (districts), with a RR of 1.90. The secondary cluster included only one county (Ningjin county in Dezhou city) with a RR of 2.57.

The spatial scan analysis showed that four significant clusters of pancreatic cancer were found in urban areas in Shandong (Table 1 and Fig. 2c). The most likely cluster covered 23 counties (districts) in the northeastern region, and the RR of this area was 2.31 times higher than that of the other areas. The other three clusters were distributed in the northern and southern regions, with RRs of 2.49, 2.86 and 1.84, respectively. After adjusting for age, only two clusters were found (Table 1 and Fig. 2d). The most likely cluster covered 23 counties (districts) in the northeastern region, and the RR of this area was 1.94 times higher than that of the other areas. The secondary cluster was distributed in two counties in the southern region, with a RR of 1.91.

Only one cluster was detected in rural areas in Shandong (Table 1 and Fig. 2e). The most likely cluster covered the eastern, northern and central regions, with a total of 65 counties (districts) and a RR of 2.32 compared to the other areas. After adjusting for age, the spatial distribution was consistent with that before the adjustment for age (Table 1 and Fig. 2f).

## Discussion

Pancreatic cancer has a very high mortality rate with a 5-year survival rate of less than 5%^23^. The mortality of pancreatic cancer has shown an increasing trend in China^7^. Therefore, exploring the spatial distribution of pancreatic cancer mortality is an important strategy to reduce the burden of pancreatic cancer. This study detected the geographic distribution and risk clusters of pancreatic cancer mortality at the county level in Shandong Province.

Consistent with previous studies^24,25^, our results showed that the mortality rate of pancreatic cancer displayed an increasing trend from the western region to the eastern region. In this research, relatively high mortality rates were mainly concentrated in some counties in the eastern and northern regions in Shandong, while relatively low rates were focused in the central and western regions. One reason for these results is that the occurrence of pancreatic cancer may be related to socioeconomic factors. In China, the eastern region is more developed than the western region, and this pattern is similarly in Shandong. According to the 2013 Shandong Statistical Yearbook^26^, Gross Domestic Product of the eastern region in Shandong, including Qingdao (7302.11 billion), Yantai (5281.38 billion), Jinan (4803.67 billion) and Weifang (4012.43 billion) are higher than those of the western region including Heze (1787.36 billion), Zaozhuang (1702.92 billion) and Laiwu (631.41 billion); the Household Consumption Expenditure of Weihai (25660 yuan), Qingdao (20659 yuan), Jinan (22755 yuan), Dongying (19370 yuan) and Yantai (17194 yuan) are higher than those of Heze (10662 yuan), Liaocheng (10819 yuan), Dezhou (12310 yuan) and other western regions. Overall, based on these economic data, we can see that the eastern region in Shandong is relatively more developed than the western region. Numerous studies have demonstrated the effects of socioeconomic factors on pancreatic cancer mortality^27–29^. One study conducted at the global level explored the association of pancreatic cancer mortality with socioeconomic development and productivity across different countries^29^. The results showed that the mortality rate increased according to Human Development Index (HDI) (men: r = 0.67; women: r = 0.72), and Gross Domestic Product (GDP) levels (men: r = 0.23; women: r = 0.28, all p < 0.05). In addition, the higher mortality in the eastern region might be related to the ageing population and lifestyle factors, which were the strongest risk factors for pancreatic cancer^30–33^. These risk factors were more prevalent in the developed region, leading to a higher mortality rate of pancreatic cancer in the developed eastern region.

The spatial scan analysis revealed four significant clusters of pancreatic cancer mortality, and the most likely cluster covered the northeastern, northern and central regions. After adjusting for age, the most likely cluster also covered the southeastern region in addition to the above regions. This also suggested that the ageing population may have an impact on the distribution of pancreatic cancer mortality. The identification of the risk clusters of pancreatic cancer mortality can provide a basis for further exploring the geographical and environmental factors of the disease and formulating feasible prevention and control measures for pancreatic cancer regionalization.

We classified areas of Shandong Province as urban/rural based on townships/subdistricts to explore the spatial distribution and clusters of pancreatic cancer mortality, respectively. Previous studies demonstrated that the geographical distribution of pancreatic cancer mortality in urban areas was not completely consistent with that in rural areas^3,10–12^. Our study also showed the distribution difference between the urban and rural areas, though it was not obvious. However, we observed that the average mortality in urban areas (4.99 per 100,000) was significantly higher than that in rural areas (3.51 per 100,000), suggesting that the occurrence of pancreatic cancer may be related to lifestyle and dietary factors. Diabetes was associated with an increased risk of pancreatic cancer^34,35^. According to a 2010 Chinese diabetes survey^36^, the number of patients with diabetes in urban areas was greater than that in rural areas. Therefore, the mortality rate of pancreatic cancer in urban areas was higher than that in rural areas. Additionally, in recent years, population ageing and the westernization of lifestyles in urban areas have also increased the risk of pancreatic cancer^37–39^.

Some limitations of this study should be noted. First, at the beginning of the study, the official population data for 2013 were not available due to a lag in demographic data, so the total population between 2010–2012 was used as an estimate of the total population between 2011–2013. Second, we evaluated the spatial distribution of pancreatic cancer mortality in Shandong from 2011 to 2013, which is a very short period. Further studies with longitudinal data are needed to explore the spatial distribution of pancreatic cancer during a long surveillance period. Third, certain potential factors such as socioeconomic factors might be associated with the clusters of pancreatic cancer, but we were unable to obtain any relevant information from the official data. Future studies should be conducted to determine the impact of these potential factors on the clusters.

## Conclusion

Our study explored the geographic distribution and clusters of pancreatic cancer mortality in Shandong Province, China. The results showed an increasing trend from the western region to the eastern region. Moreover, we also observed differences in the geographic distribution and risk clusters between urban and rural areas. These findings can provide a basis for targeted and effective measures for controlling pancreatic cancer in different regions.

## Data Availability

We acquired the data on pancreatic cancer death through official surveillance of the Shandong Death Registration System (SDRS). All data generated and analysed during the course of this study are available from the corresponding author upon request.
